# Post-Traumatic Hypopituitarism—Who Should Be Screened, When, and How?

**DOI:** 10.3389/fendo.2018.00008

**Published:** 2018-02-02

**Authors:** Mark Quinn, Amar Agha

**Affiliations:** ^1^Department of Endocrinology, Beaumont Hospital and School of Medicine, Royal College Surgeons in Ireland, Dublin, Ireland

**Keywords:** traumatic brain injury, post-traumatic hypopituitarism, hypopituitarism, pituitary injury, growth hormone deficiency, ACTH deficiency, incidence of post-traumatic hypopituitarism, screening for post-traumatic hypopituitarism

## Abstract

Traumatic brain injury (TBI) remains a major, global public health concern. Over the last 15 years, a significant body of evidence has emerged demonstrating that post-traumatic hypopituitarism (PTHP) is a common and clinically significant consequence of TBI. Non-specific symptomology and the lack of an agreed approach to screening for PTHP has led to significant under-diagnosis of this debilitating disease. In this review, we will discuss the frequency and clinical significance of acute and chronic PTHP as described in the current literature highlighting the evidence base for screening and hormone replacement in these patients. We will also offer a pragmatic approach to identifying relevant anterior pituitary dysfunction after TBI and a follow-up strategy for those patients. Specific controversies and remaining unanswered questions will be addressed.

## Introduction

The true global incidence of traumatic brain injury (TBI) is difficult to fully ascertain; a recent meta-analysis suggested that the incidence is increasing and affects 134–618 persons per 100,000 per year with the highest incidence appearing in adult men (1). This makes TBI the most common cause of death and disability in young adults living in industrialized countries (2) and as such it presents a major public health concern.

However, the reported incidence of TBI varies wildly depending on numerous geographical and demographic factors. Most TBIs are mild and are not admitted to hospital (3). As a result, the vast majority of the literature on the subject relates to patients who required hospital admission, typically with moderate or severe TBI. The resultant underestimation of the true incidence of TBI has led to it being described as a “silent epidemic.”

Over the recent 15 years, numerous research studies have documented a high rate of pituitary gland dysfunction following moderate or severe TBI with evidence that post-traumatic hypopituitarism (PTHP) contributes significantly to the morbidity and possibly mortality in those patients (4). PTHP can masquerade as a post-concussional syndrome and can be overlooked leading to delayed recovery and impaired rehabilitation (5). Therefore, screening patients for PTHP should be an important part of the standard care provided following severe or moderate TBI.

This review will discuss the evidence supporting the need for screening of anterior pituitary function after TBI and our approach to the endocrine assessment and management of these patients.

### PTHP—Epidemiology and Natural History

Pituitary damage was first recognized as a potential outcome of head injury in 1918 when it was reported in an individual with a fractured base of skull (6). In the decades that followed, it was generally considered to be a rare complication of TBI. A review in 2000 reported only 367 known cases in the literature (7). However, over the last 15 years, there has been numerous retrospective and prospective studies showing a high rate of PTHP among long-term survivors of TBI (Table 1). In one meta-analysis, the pooled data from 19 studies showed a prevalence of 27% (8). The variations in the reported frequency of PTHP reflect patient selection, timing of testing, and methodological heterogeneity in the ascertainment of hypopituitarism. Among the pituitary hormone deficiencies, growth hormone (GH) deficiency, as with other forms of hypopituitarism, appears to be the commonest deficiency in those who were tested 6 months or more following the event (Table 1).

**Table 1:** A summary of the main studies assessing the frequency of post-traumatic hypopituitarism.

Study	Patient number	Male/female	Median age at traumatic brain injury (TBI)	TBI severity	Timing of test post TBI	LH/FSH def.	Growth hormone def.	TSH def.	ACTH def.	Diabetes insipidus
Personnier et al. (9)	87	60/27	5.9 (0.2–14.4)	Severe	6–18 months	–	31%	2.3%	1.1%	–
Baxter et al. (10)	19	19/0	26.7 (26.1–30.9)	Moderate–severe	2–48 months	5.3%	15.8%	0%	10.5%	0%
Hannon et al. (11)	32	–	–	Moderate–severe	6–24 months	3.1%	18.8%	0%	18.8%	–
Hannon et al. (11)	100	85/15	33 (18–75)	Moderate–severe	1–22 days	–	–	–	78%	51%
Kozlowski Moreau et al. (12)	55	46/9	36.1	Mild–severe	At least 12 months	–	40%	21.8%	27.3%	–
Schneider et al. (13)	78	52/26	36.0	Mild–severe	3 months	32.4%	9.1%	7.8%	19.5%	–
Schneider et al. (13)	70	47/23	35.7	Mild–severe	12 months	28.6%	14.3%	4.3%	12.9%	–
Agha et al. (14)	102	85/17	28 (15–65)	Moderate–severe	6–36 months	11.8%	10.7%	1.0%	12.7%	–
Tanriverdi et al. (15)	52	43/9	35.9 (17–65)	Mild–severe	12 months	41.6%	20.4%	5.8%	9.8%	–
Aimaretti et al. (16)	70	50/20	39.31	Mild–severe	3 months	17.1%	38.5%	5.7%	8.5%	4.2%
Aimaretti et al. (16)	70	50/20	39.31	Mild–severe	12 months	11.4%	38.6%	5.7%	7.1%	2.8%
Agha et al. (17)	102	85/17	28 (15–65)	Moderate–severe	1–24 days	–	–	–	–	21.6%
Agha et al. (17)	102	85/17	28 (15–65)	Moderate–severe	6–36 months	–	–	–	–	6.9%

Prospective longitudinal studies have shown that pituitary dysfunction occurs early after acute TBI. The most clinically significant abnormalities in the acute phase of TBI are ACTH-cortisol deficiency and salt and water imbalance (11, 17). Some of these early abnormalities are transient and recover fully within days or weeks after TBI while new pituitary hormone deficiency may become apparent in the post-acute phase (13, 14). The symptoms of hypopituitarism can be subtle (18) and are summarized in Table 2.

**Table 2:** A summary of common symptoms and findings associated with specific pituitary hormone deficiencies.

Hormone deficiency	Symptoms	Findings
ACTH	Life-threatening adrenal crises especially during acute illness, weakness, lethargy, weight loss	Hypotension, hypoglycemia, hyponatremia, hypercalcemia, anemia
Growth hormone	Decreased energy, low mood, neuropsychiatric symptoms, poor quality of life	Decreased lean body mass, increased fat mass, altered metabolic profile, decreased exercise capacity, reduced BMD
LH/FSH	Oligo/amenorrhea, mood disturbances, decreased libido, seating, erectile dysfunction	Decreased lean body mass, reduced secondary sexual characteristics, infertility
TSH	Fatigue, weakness, weight gain, constipation, neuropsychiatric problems	Myopathy, bradycardia, skin/hair changes, hypothermia
Vasopressin	Polyuria, polydipsia, nocturia, incontinence	Dehydration, hypernatremia

## Pathophysiology

Mechanisms of injury including vascular injury to the hypothalamus, stalk, or the pituitary gland resulting in infarction, direct traumatic injury to the pituitary from base of skull fractures or secondary insults from, hypoxia, hypotension, or raised intracranial pressure (19) have all been hypothesized. It is likely that a vascular injury is the commonest mechanism as autopsy studies have demonstrated pituitary infarction in up to 43% of cases of fatal TBI (20).

The relationship between the severity of the head injury and the risk of hypopituitarism remains controversial. Traditionally, the clinical severity of TBI is measured by the post-resuscitation and pre-intubation Glasgow Coma Scale (GCS) Score. A score of 3–8 indicates severe, 9–12 moderate, and 13–15 mild injury. In patients with moderate or severe TBI, the scores do not seem to predict the risk of PTHP (8) but it is important to emphasize that this scale is limited by significant inter-observer variability and that the GCS scores can fluctuate over time. Some authors have suggested that findings on computed tomography should be factored in to the initial assessment when determining severity of TBI. They have hypothesized that significant pathological findings like cerebral edema or hemorrhage on cranial imaging may infer a more severe TBI. The data on this is limited with one study demonstrating a correlation between diffuse brain swelling and PTHP (19) and another finding no such association (14).

The data for mild TBI and hypopituitarism are limited (3). While some studies showed a significant association (21), one excellent large study showed a very low incidence of PTHP in mild TBI cases (22).

## What is the Clinical Significance of PTHP?

In patients with TBI, acute-phase cortisol deficiency is potentially life threatening and a number of studies have shown an association between increased morbidity and mortality in acute post-traumatic hypoadrenalism (11, 23).

Long-term adult GH deficiency is associated with decreased quality of life (24), reduced lean body mass (25), reduced bone mineral density (26), and impaired cardiac function (27). The sequelae of sex steroids, ACTH, and TSH deficiencies are well known (Table 2). Recent data seem to confirm that anterior pituitary hormone deficiency has a negative impact on functional outcome at 6 months post TBI as assessed by mini-mental state exam and functional independence measure scores (4). Other studies have found a correlation between PTHP and unfavorable metabolic and body composition profiles with associated lower quality of life scores (28, 29) as well as decreased exercise capacity (30) and neuropsychiatric complications (31).

## Who to Screen?

Screening every patient with a TBI for PTHP is clearly not feasible. This approach does not meet the criteria for an appropriate screening tool due to the high cost and the complexity of testing which often requires dynamic and sometimes repeat assessments. The most robust data with regard to incidence of PTHP are in those patients with moderate or severe TBI (14). We therefore do not routinely screen patients with mild TBI for pituitary dysfunction unless there are specific symptoms suggestive of hypopituitarism (21, 22). Features that seem to confer an increased risk of PTHP include diffuse axonal injury on brain imaging, prolonged admission to ICU, basal skull fracture, and increasing age (32).

Screening for mild cases of TBI remains controversial. Schneider et al. demonstrated a high rate (16.8%) of PTHP following a mild TBI (8) although most of the endocrine abnormalities on mild TBI cases were not confirmed by second dynamic tests. In one well-executed study, the frequency of PTHP after simple mild TBI was found to be very low (22). However, it has been suggested that “complicated” cases labeled as mild TBI should also be included in the screening process (33). Features of a complicated mild TBI as described in this review included those patients admitted to hospital for >24 h, patients admitted to the ICU or those requiring neurosurgical intervention, patients suffering pituitary dysfunction within 2 weeks of their TBI, or patients with new anatomical changes on their brain scans. Therefore, selected cases of complicated mild TBI could also be offered screening but this will clearly have considerable resource implications.

## How and When to Screen

### Acute-Phase Assessment

There is currently no evidence to support the treatment of acute GH, thyroid hormones or gonadotropins deficiencies, or hyperprolactinemia in the acute phase following TBI. In addition, interpretation of the results in the acute phase is difficult. Therefore, early assessment of these axes is unnecessary.

**Figure 1:**
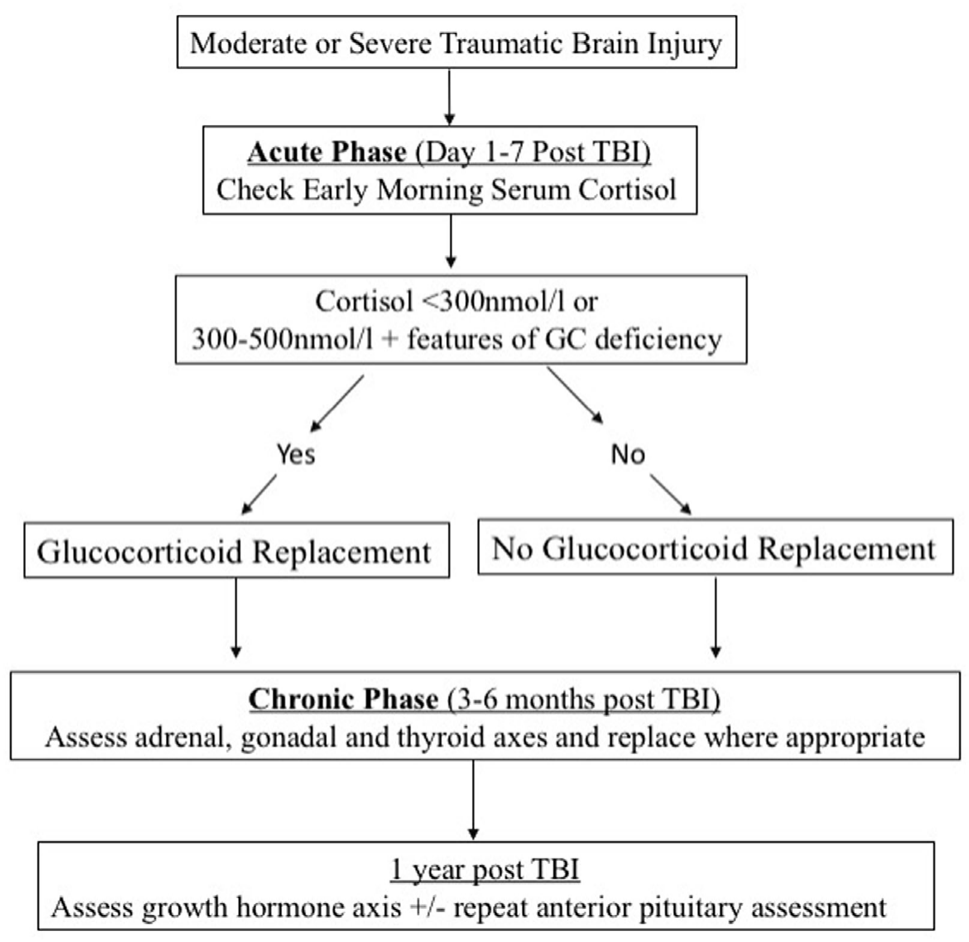
Algorithm for the screening and management of post-traumatic hypopituitartism.

Assessment of acute anterior pituitary dysfunction following TBI should focus on ACTH deficiency because of its potential contribution to the acute morbidity and mortality (11) in this patient cohort. Under normal circumstances, a dynamic test of the adrenal axis (such as the insulin stress test or the corticotrophin test) would provide the most accurate assessment. However, these dynamic tests are either inappropriate (the insulin stress test) or unreliable (the corticotrophin test) in the acute setting of TBI. For this reason, serial morning cortisol levels can be checked as a guide to possible ACTH deficiency. An acute-phase cortisol level below 300 nmol/l is suggestive of adrenal insufficiency (34) and treatment with glucocorticoids should be instituted (Figure 1). Levels of 300–500 nmol/l should be interpreted in the clinical context. Features suggestive of adrenal insufficiency as listed in Table 2 should alert the clinician and treatment with GC replacement may be warranted.

### Chronic Phase Assessment

All patients with moderate and severe TBI should have an assessment of their adrenal, thyroid, and gonadal axes in the post-acute phase, usually at 3–6 months post TBI. The choice of the dynamic test for adrenal reserves will depend on the preference of the endocrinologist but we prefer the corticotrophin test (Synacthen test) as a simple and reliable test (35). Also, thyroid function tests (free T4 and TSH) and sex steroids with corresponding gonadotrophins levels (and menstrual history in premenopausal women) should be assessed. If a deficiency is detected, we recommend replacement as appropriate and repeating the assessment at 1 year as early deficiencies may recover (36).

Early GH deficiency post TBI can be transient (36). Assessment of the GH axis is also more complex due to the need to perform dynamic testing which is often only available in specialist centers. For these reasons, we recommend deferring GH assessment until 1 year post TBI. Assessment of GH reserves should ideally be done with the gold standard insulin tolerance test provided no contraindications (especially seizures and heart disease) are present. Other dynamic tests for GH can also be used (37) (Figure 1).

## Conclusion

Traumatic brain injury is one of the world’s leading causes of morbidity and mortality among young men. PTHP is now recognized as a common, but often underdiagnosed, complication which may contribute significantly to the morbidity and possibly the mortality following moderate and severe TBI. Endocrine evaluation and management should be part of standard multidisciplinary care for these patients. Among patients with severe and moderate TBI, acute cortisol deficiency should be diagnosed and managed promptly while a more comprehensive assessment of the pituitary function should be undertaken in the post-acute phase. Randomized clinical trials examining the effect of GH replacement in patients with post-traumatic GH deficiency are needed to assess the potential impact on recovery, rehabilitation, and quality of life.

## Author Contributions

MQ carried out a detailed literature review on the topic of post-traumatic hypopituitarism and created various drafts under the title “Post-traumatic hypopituitarism—who should be screened, when, and how?” AA corrected each draft and provided additional literature which added to the scope of the review.

## Conflict of Interest Statement

The authors declare that the research was conducted in the absence of any commercial or financial relationships that could be construed as a potential conflict of interest.
